# Functional Gastrointestinal Diseases and Dietary Practices among Pakistani Children—A Schools Based Cross-Sectional Study

**DOI:** 10.3390/diseases10040103

**Published:** 2022-11-16

**Authors:** Zoha Imtiaz Malik, Muhammad Farooq Umer, Khizar Nabeel Ali, Ayesha Babar Kawish, Muhammad Arshed, Shumaila Zofeen, Awais Farid

**Affiliations:** 1 Al-Shifa School of Public Health, Rawalpindi 43600, Pakistan; 2 College of Dentistry, King Faisal University, Al-Ahsa 31982, Saudi Arabia; 3 Department of Community Health, Faculty of Medicine and Health Sciences, University Putra Malaysia (UPM), Serdang 43400, Selangor, Malaysia; 4 School of Public Health, Xi’an Jiaotong University, Xi’an 710049, China; 5 Division of Environment and Sustainability, Hong Kong University of Technology, Hong Kong, China

**Keywords:** diet, functional gastrointestinal diseases, FFQ, gut disorders, nutrition, Rome IV criteria, school children

## Abstract

Background: Functional gastrointestinal diseases (FGIDs) are an important yet highly under explored area among public health issues. FGIDs’ complex etiology makes them of interest along with their prevalence in children steadily increasing, especially in the developing world. We aimed to determine the burden FGIDs pose on school-going children, and to determine its association with the dietary intake patterns in Pakistani children. Methodology: The study included 385 school-children from public and private schools in Pakistan through multistage random sampling, from March to August 2022. We used the Food Frequency Questionnaire (FFQ) and Rome IV Criteria for a comprehensive exploration of the issue. Associations between the FGIDs and dietary factors were analyzed using chi-square and Fischer’s exact tests in SPSS version 26.0. Results: Females constituted 77.4% (*n* = 298) of all respondents, while 44.9% (*n* = 173) of the total reported a family history of gastrointestinal diseases. FFQ analysis showed varying consumption frequencies for different food groups. Functional abdominal pain and irritable bowel syndrome (IBS) were the highest reported FGIDs with a prevalence of 38.7% (*n* = 149) and 24.9% (*n* = 96), respectively. Statistical associations were found between different FGIDs and gender, age, household income, family members, and dietary variables such as fruit, vegetable, beverage and pulse consumption. Conclusion: FGIDs were found to be associated with a number of socio-demographic and dietary factors which calls for small scale and large scale attention to the issue. Results from the current study and further studies may help develop guidelines to manage these disorders in Pakistan.

## 1. Introduction

In recent years, complaints of functional digestive issues have initiated investigations of their causes, showing associations with gastrointestinal carcinomas, intestinal blockage, ulcers, reflux diseases, and others [1]. 

Research on gastrointestinal disorders over the years has suggested functional gastrointestinal diseases (FGIDs) as the most common types of gastrointestinal diseases, especially in infants, children, and adolescents. As of 2021, 40% of the entire world’s population has some kind of gastrointestinal disease, with the prevalence higher in females than males [1], while 27% of infants, 40.5% of toddlers, 9.9% of children, and 27.5% of adolescents are affected [2]. The United States of America has a prevalence rate of 11% for chronic gastrointestinal diseases, with the geriatric population—those aged 65 years or older—showing a 35% prevalence rate for these disorders [1] and 23.1% children reporting at least 1 digestive disorder [3]. A study in the UK found 81% of the participants to have at least one gastrointestinal issue, with flatulence being the highest reported one [4] and 25% of the infant and child population suffered from abdominal pain and gut disorders [5]. Data from Middle-Eastern countries depict a slightly higher gastrointestinal disease prevalence in contrast to that of the developed countries, as evidenced by a prevalence of 30.4% among children and adolescents aged 4 to 18 years [6]. Data from Egypt show a 12–29% prevalence in the general child population, and 25% prevalence in school-children is reported in Jordan [7]. Asia’s prevalence rates are a mix of high and low with India reporting a 10% prevalence of functional gastrointestinal diseases in adolescents aged 10 to 17 years [8] while China has a prevalence as low as 5.4%, mainly because of the well-developed healthcare system. 

Like the rest of the world, FGIDs are also an emerging issue in Pakistan though the problem remains underexplored, as evidenced by the lack of data available. The countrywide prevalence of all FGIDs in Pakistan remains unknown. Research has reported a 33.2% prevalence for irritable bowel syndrome (IBS) which is the most common FGID in the country [9]. In college students, functional IBS had a prevalence of 15.5%, while stress, sleep disturbances, and a positive IBS family history were all found to be significantly related to the diagnosis [10]. Female respondents depicted more severe symptoms, while a higher percentage of male respondents used medications to relieve constipation [11]. About 37.5% of the pediatric patients, aged 2–14 years, visiting a tertiary care setting were seen to be suffering from functional constipation [12]. 

An array of factors play a role in the etiology and management of FGIDs, namely bio-psychosocial aspects, genetics, gender, ethnicity and culture. Our focus in this study remains on the dietary factors that might play a role in FGID development, particularly in school children. Eating habits such as irregular meals, eating close to bedtime, eating foods lacking quality, and consuming foods that are rich in sugar, fat, and lactose may cause digestive problems, while a low FODMAP diet along with fiber and probiotics helps maintain a healthy gut microbiota [13]. Through the years, various studies have pointed out the relation between diet and gut functioning, and its influence on both the pathogenesis and management of gastrointestinal issues [14]. Among children of school-going age, around 93% suffer from some form of digestive issue related to food intolerances and/or poor dietary patterns. Gluten and lactose intolerance are the most common causes of digestive issues in this age group [15]. However, the association of diet with the prevalence of functional gastrointestinal diseases in school-going children in Pakistan remains unexplored, thereby constituting the rationale for this study. 

Functional gastrointestinal diseases are a source of morbidity and reduced health-related quality of life in children and adolescents. FGIDs contribute not only to a child’s physical and mental stress, but also make them suffer academically. Students with a history of abdominal pain and discomfort have been observed to be a year behind their peers, as well as portraying reading and comprehension difficulties [16]. Additionally, poor dietary habits have been linked to a drop in students’ academic performance. The study revealed that, for every level of food insecurity, the GPA for students dropped by 0.12 points [17], indicating the vital relationship between dietary consumption and school performance. 

Despite being an emergent public health issue, functional gastrointestinal diseases, especially their occurrence in the school-going child and adolescent population, are overlooked in Pakistan. The current study is an effort to determine the burden of functional gastrointestinal diseases in school-going children as well as find the association between their dietary intake patterns and FGID prevalence. This will help identify the burden of FGIDs in this section of the population and help determine if diet plays any role in their occurrence at a young age. The findings may serve to be beneficial for future management, and dietary recommendations relevant to functional gastrointestinal diseases in this age group.

## 2. Methodology

Our study was a cross-sectional, school-based one, conducted in the two metropolitan cities of Pakistan from March to August 2022. 

Sample selection 

The sample size for the study was calculated to be 385 respondents by the following equation:

N = (z^2^ × P × Q)/E^2^

where z is the confidence level at 95% (standard value of 1.96), P is the estimated prevalence or proportions in previous studies, Q is 1-P, and E = margin of error. The prevalence of FGIDs was assumed to be 50%, which when put in the formula gives a total sample size of 384 participants, with a confidence interval of 95% and a subsequent margin of error of 5%. 

The inclusion criteria allowed children aged 12 years and above, and with no physical or mental illnesses to affect their responses, to be a part of the study. The response rate for the study was 100%. 

The schools were selected via multi-stage random sampling by firstly obtaining lists of schools from the public and private school federations. Schools were then selected from each list by employing the lottery method. A total of 8 schools were selected to meet the sample size, with one public and three private institutes from each city. The reason why two public schools were selected as opposed to six private ones include the greater number of private schools in both cities as compared to public schools, and the high number of students enrolled per class in public schools in comparison to those enrolled in private schools. After selection of schools, classes with students aged 12 years and above were selected via systematic sampling and the students from each class were also selected via systematic sampling, using the attendance sheet. 

**Figure 1:**
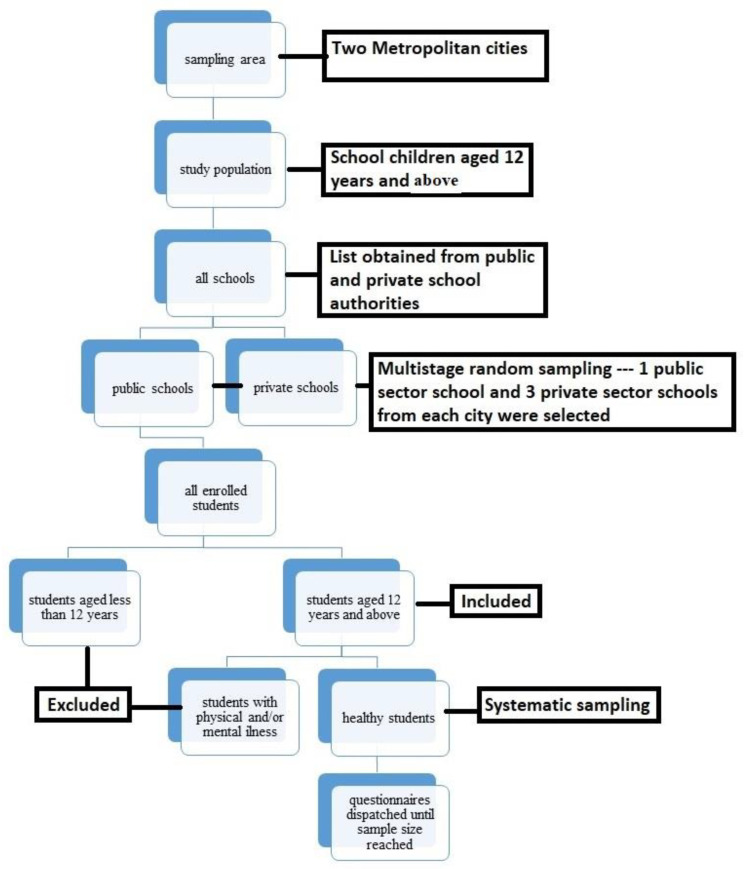
Flow Sheet Diagram for Sampling.

Official permission letters to conduct the study were obtained from each school, with informed consent from the children’s legal guardians. Data was collected from these students via questionnaires consisting of three parts; socio-demographics, dietary information, and gastrointestinal symptom assessment (Figure 1).

(a) *Socio-Demographics*

Data on various socio-demographics such as age, gender, grade of study, family type, family size, paternal relationship and occupational status, and family income, were collected. Additionally, questions addressing eating habits, such as frequency of dining out and bringing lunch from home to school, physical activity, such as frequency of playing sports or exercising, were also included. Family history regarding gastrointestinal issues was also asked in an effort to gauge the genetic aspects of functional gastrointestinal issues.

(b) *Dietary Information*

A Food Frequency Questionnaire (FFQ) was used to gather information regarding the eating habits of respondents. The questionnaire was an adapted version of the Youth Adolescent Food Frequency Questionnaire, and was modified to fit the eating patterns of Pakistani children. Two experts adapted the FFQ and then pilot tested it for any potential issues. The Cronbach alpha for the adapted version was found to be 0.809. The FFQ contained foods and drinks from 8 food groups, namely cereals and grains, fruits, vegetables, meat and meat products, legumes, pulses and lentils, dairy and dairy products, snacks and deserts, and beverages. The frequency of consumption was divided into 6 categories, never, less than once a week, once a week, 2–3 times a week, 4–6 times a week, and every day.

(c) *Rome IV Criteria*

Over the years, the Rome criteria have been utilized as a diagnostic and categorization tool for these disorders, in all age groups including school-going children. The latest version is the Rome IV criteria developed in 2016 which now include 2 new diagnostic categories for children above 4 years of age, including functional nausea and functional vomiting [18]. The IBS symptoms were changed from ‘pain that goes away after passing a stool’ to ‘pain that is related to defecation’, and functional dyspepsia symptoms were made specific to get a more precise diagnosis [19]. In addition to these changes, several other inclusions were made such as functional dyspepsia diagnosis based on a minimum frequency of symptom occurrence, and the addition of functional abdominal bloating when bloating or distention is the primary symptom [20]. 

Several school based studies in the past have used different versions of the Rome criteria to determine the burden of FGIDs, including a Chinese study using the Rome IV criteria to diagnose 27.3% children with at least 1 FGID [21]. Researchers in Saudi Arabia used it to report a 3.1% and 4.7% prevalence of functional abdominal pain and functional constipation, respectively [22]. The Rome IV questionnaire reported 30.4% of Egyptian school children to have one FGID, while irritable bowel syndrome was the most prevalent with a prevalence of 11.6% [6]. 

To assess the gastrointestinal symptoms in children in this study, the Rome IV Diagnostic Questionnaire for Pediatrics FGIDs (R4PDQ) for ages 10 and up (Child: Self-report form) was employed. The questionnaire is divided into 5 sections including: Section A—Pain and uncomfortable feelings above the belly button, Section B—Bellyaches and abdominal pain around and below the belly button, Section C—Bowel movements, Section D—Nausea and vomiting, and Section E—Other symptoms. All of these sections comprise a series of questions regarding gastrointestinal related symptom occurrence and frequency. These questions are designed to diagnose functional gastrointestinal disorders including functional dyspepsia (post-prandial distress syndrome and epigastric pain syndrome), irritable bowel syndrome (IBS), abdominal migraine, functional abdominal pain, functional constipation, functional nausea, functional vomiting, cyclic vomiting syndrome, adolescent rumination syndrome, and aerophagia.

Permission was obtained from the Rome Foundation to use the questionnaire after signing an official license. No changes were made in the questionnaire, and it was used in its entirety as our study aimed to assess the prevalence of all the FGIDs. The Cronbach alpha for the Rome IV Diagnostic Questionnaire was found to be 0.795. 

### Statistical Analysis

The data collected were analyzed via chi square goodness of fit test and Fischer’s exact test, where applicable, using the SPSS version 26. The cut-off p value for significant results was set at 0.05, with a confidence interval of 95%.

## 3. Results

(a) *Descriptive Results*

Based on gender, 87 (22.6%) of the total respondents were males and 298 (77.4%) were females, out of the total sample of 385 students (Table 1); 50.9% (*n* = 196) of the total respondents resided in Rawalpindi. The age-wise distribution of the students included 38.2% (*n* = 147) in the age group of 12–13 years, 43.1% (*n* = 166) in the age group 14–15 years, and 18.7% (*n* = 72) of age 16 years and above. The frequency of students dining out once a week was seen to be 37.1% *(n* = 143), while 54.8% (*n* = 211) admitted to bringing lunch from home less than once a week. 

Results obtained from the Food Frequency Questionnaire (FFQ), (Table 2) indicate that 34% (*n* = 131) of students consumed cereals once a week, and 10.4% (*n* = 40) consumed them 4–6 times a week. Fruits were consumed once a week by 31.2% (*n* = 120) of students, and every day by 9.1% (*n* = 35) of the children. The consumption frequency for vegetables was less than once a week by 33.5% (*n* = 129) of the 385 participants, 2–3 times per week by 16.9% (*n* = 65), while 2.1% (*n* = 8) never consumed any vegetables. Of the 385 participants, 43.1% (*n* = 166) consumed meat and meat products once a week, while 3.4% (*n* = 13) consumed some form of meat products every day. Pulses and legumes were consumed less than once a week by 29.1% (*n* = 112) of the participants and 4–6 times a week by 6.8% (*n* = 26) of the 385 interviewed children. Dairy and dairy products were consumed 2–3 times a week by 30.6% (**n** = 118) of students while 9.4% (*n* = 36) of the students consumed them every day. Different types of snacks were consumed 4–6 times a week by 27.8% (*n* = 107) of the study participants and 12.5% (*n* = 48) were reported to consume them daily. Thirty-three percent (*n* = 127) of students consumed beverages 2–3 times a week, while 9.6% (*n* = 37) had them every day. 

The highest reported FGID was functional abdominal pain (38.7%), followed by irritable bowel syndrome (24.9%), while aerophagia (1%) and functional vomiting (0.5%) remained the least reported (Table 3). 

### Associative Relationship between Functional Gastrointestinal Disease Prevalence and Various Socio-Demographics and Dietary Factors

The chi-square test of association found significant associations between several functional gastrointestinal diseases and socio-demographic factors, and food groups, the most prominent of which are included in Table 4. 

No significant association between gender and PPD was found (*p* = 0.58) while physical activity was significantly associated with the disorder (*p* = 0.01). Fruits and vegetables consumption was also significantly related with PPD (*p* = 0.007, *p* = 0.03) respectively. 

Significant association between epigastric pain and gender was found (*p* = 0.008). Household income also showed reported a significant association between the two variables (*p* = 0.002). Dining out and bringing a home lunch were significantly related with epigastric pain (*p* = 0.023, *p* = 0.04), respectively. 

Irritable Bowel Syndrome (IBS) showed a significant association with gender (*p* = 0.001), and physical activity (*p* = 0.05). 

Functional abdominal pain and age showed statistically significant results (*p* = 0.01). Gender and physical activity were also statistically significant (*p* = 0.001) and *p* = 0.001), respectively, while pulses consumption were also found to be statistically significant (*p* = 0.05). 

There was a statistically significant association between functional nausea and number of family members, as evidenced by the chi-square test of association (*p* = 0.04).

The chi-square test of association found abdominal migraine to be statistically significantly associated with home-based lunch (*p* = 0.04), and beverage consumption (*p* = 0.04). No other socio-demographic or dietary variable was found to be significantly associated with the disorder. 

Adolescent rumination syndrome was only found to be significantly associated with pulses consumption (*p* = 0.05). 

However, functional constipation, and aerophagia were not found to have statistical associations with any of the socio-demographic or dietary variables. 

## 4. Discussion

The current study aimed to assess the prevalence of Functional Gastrointestinal Diseases (FGIDs) and dietary habits of school children. FGIDs are a group of lesser studied and understood disorders, especially in countries like Pakistan, which was the basis for conducting this research. All of the FGIDs acknowledged by the Rome Foundation in their Rome IV criteria were assessed in the study population to develop a picture of their prevalence in the school child population group. The results indicated a varying prevalence of different FGIDs, with functional abdominal pain and IBS being the highest reported disorders, at a prevalence of 38.7% (*n* = 149) and 24.9% (*n* = 96), respectively. Only 4 cases of aerophagia, 2 cases of functional vomiting, and no cases of cyclic vomiting were reported. 

Previous studies have also found a significant relation between IBS and gender, with the disorder being more common in females than males, as evidenced by a female-to-male ratio of 2–2.5:1 [23]. This is consistent with our study’s finding that gender is highly significantly associated with functional IBS (*p* = 0.001), with female respondents constituting 89.6% of the total cases of the disorder. Functional IBS has also been linked with physical activity in previous studies that reported positive long-term effects of physical activity-related interventions in IBS patients. One such study reported an improvement in five out of nine IBS-specific dimensions [24]. Our study also found a significant association between the occurrence of IBS and engaging in physical activity or sports (*p* = 0.05), thereby adding to the evidence promoting the relation between exercise and IBS prevalence. IBS was also found to be significantly associated with the school area (rural or urban), with 52.7% of the total IBS cases being from schools present in the rural areas (*p* = 0.04). However, a study in Bangladesh found the prevalence to be higher in urban settings and was also reported to be much lower as compared to that in other Asian countries [25]. Another study reported that the reason for a low prevalence of IBS in rural areas could be because of the lack of diagnostic services present as a result of poor healthcare [26].

Several studies associated abdominal pain prevalence with age; while 9 of these studies found no association between the two variables, four of these studies reported a decreased prevalence with age, which is similar to the findings of this study [5]. The highest prevalence of functional abdominal pain (44.3%) was reported in the age group 12–13 years, after which it gradually started to decrease for the remaining ages. Additionally, the role of diet as a causative or contributing factor in functional abdominal pain has been under-explored and the studies that have focused on dietary aspects of the disorder have not reached conclusions on an effective dietary solution [27]. The current study has also not found any statistically significant association between any of the food groups, except pulses and lentils (*p* = 0.05). 

The study found the number of family members in a child’s family to be statistically significantly associated with functional nausea occurrence (*p* = 0.04). This can be related to the fact that larger families have been associated with increased stress and anxiety, as evidenced by a study suggesting that intra-family conflict and economic pressures arising from living with a larger number of family members contribute to the children’s social experience and psychological stress [28]. No other socio-demographic or dietary variable in our study was found to be significantly related to functional nausea. 

The pathophysiology of functional constipation is a mix of various factors but has remained ambiguous. The main contributing factors are painful bowel movements, slow transit, genetics, and the family’s child-rearing practices [29]. Our current study did not aim to explore the aforementioned aspects of the disease and found no significant association between functional constipation and socio-demographic and dietary variables included in the study. Another similar study conducted on the pediatric population in Romania concluded that those with functional constipation consumed more meat and milk products, and carbonated beverages, while the control group consumed less of these food groups and more fruits and vegetables. 

Epigastric pain and post-prandial distress syndrome were both assessed to determine the number of functional dyspepsia cases in the sample, as both these symptoms are characterized as a reliable diagnostic criterion [30]. This study also found epigastric pain to be highly and statistically significantly associated with gender (*p* = 0.008), which is consistent with the results of another study which depicted that due to the gynecological and physiological differences between the genders, women tend to suffer from epigastric pain more than men [31]. Our study also found a significant association between dining out (*p* = 0.02) and bringing lunch from home (*p* = 0.04), which depicts that eating out instead of home-based meals contributes to epigastric pain. 

Post-prandial distress (PPD) syndrome is another symptom used to diagnose functional dyspepsia and is considered a sub-type of the disease. Our study found the prevalence of PPD to be 4.9%, as compared to a 2018 study that found the prevalence to be 7.3% in the study sample. The study found no statistically significant factors associated with PPD syndrome [32], as opposed to our study finding a statistically significant association with income (*p* = 0.04), engaging in physical activity (*p* = 0.01), vegetable consumption (*p* = 0.03) and highly statistically significantly related to fruit consumption (*p* = 0.007). An intervention study designed a physical activity intervention for participants suffering from post-prandial distention and bloating, and found a significant association between PPD and post-prandial exercise, with participants reporting improvements in their symptoms [33]. These findings are consistent with our study’s results and conclude that a physical activity regimen can prove to be a more efficient method of relieving PPD symptoms as opposed to other treatment forms. Additionally, mixed fruit juices and citrus fruit juice have been seen to have a positive effect on reducing PPD, as evidenced by previous studies. Our study also found vegetables to be significantly associated with post-prandial distress syndrome; another similar study did not find such associations, however, but did conclude that certain vegetables, when consumed at least once a week, showed inverse relations with the disease, and were associated with reduced odds of the syndrome. 

Abdominal migraine was found to have a prevalence of 0.8% in our current study, which can be explained by the fact that the disorder generally affects a smaller proportion of the pediatric population with a prevalence of 0.2% and 2.1%. Abdominal migraine is triggered by foods with high amine content and containing MSG. Most beverages contain high amine content and this explains why our study found beverages to be significantly associated with abdominal migraine (*p* = 0.049). Additionally, our study found abdominal migraine to be significantly associated with not bringing home lunch which is consistent with the aforementioned study’s findings that foods that contain additives like MSG and food coloring have a role in triggering abdominal migraine symptoms [34]. Abdominal migraine was not found to be associated with any other variable in our study.

Functional vomiting was only present in 0.5% of the sample while no case of cyclic vomiting was reported. This is consistent with another study conducted on school-aged children in Columbia, which found functional vomiting to be present in 0.7% when participants were assessed using the Rome IV criteria [35]. Cyclic vomiting has an estimated prevalence of 1.9% to 2.3% and an incidence of 3.2 cases per 100,000 population. Due to the disorder being frequently misdiagnosed, there are no certain prevalence and incidence rates present [36]. This explains why no case of cyclic vomiting was detected in our study, as the sample size was much smaller than that in which a case of the disorder might be detected. Both functional and cyclic vomiting were also not significantly associated with any of the socio-demographic or dietary variables. This can be explained by the fact that cyclic vomiting is mainly triggered by psychological and physical stressors while the dietary role is limited [37]. 

Adolescent rumination syndrome had a prevalence of 1.6% in our study sample of 385, which is not an unusual statistic as the disorder is considered uncommon and is rarely reported. In another study assessing the prevalence of adolescent rumination syndrome, only 5.1% cases of the disorder were found with the occurrence being high in males [38]. Although our study found pulses consumption to be significantly associated with the syndrome, there is no evidence in previous literature to support this finding. Some common triggers for adolescent rumination syndrome include certain foods, stress, eating disorders, and physiological conditions such as abdominal wall contractions [39]. Lastly, only 4 cases (1%) of aerophagia cases were found to occur in our sample population, which is consistent with the results of a similar school-based study conducted in Indonesia; the latter study reported the prevalence to be 4.5%, but when the belching criterion was excluded from the study, the prevalence dropped to only 0.1% [40]. Another school study conducted in Sri Lanka found 7.5% of the children to be suffering from aerophagia and found a significant relationship between exposure to stress and the onset of the disorder [41]. However, our study did not find any statistically significant association between aerophagia and any of the variables. 

## 5. Conclusions

The present study is one of the initial studies that focused on determining the burden FGIDs impose on school children, and their association with dietary habits in Pakistan. This study gives an insight into the importance of investigating these disorders in school children, owing to their adverse physical, mental and academic effects on this population group. The results of the study signified significant associations of FGIDs with various socio-demographic and dietary variables such as associations between IBS and gender, functional abdominal pain and sports, and post-prandial syndrome and fruit consumption, while other FGIDs showed no association such as functional constipation. FGID etiology, development, and dietary involvement need to be further investigated to develop management strategies. The findings may prove to be beneficial when making nutrition and health-related policies for school children, at any level.

## Figures and Tables

**Table 1:** Socio-Demographic and Lifestyle Data for School Children Enrolled in the Study (*n* = 385).

Variables	Categories	Frequency in Sample	Percentage in Sample (%)
Age	12–13 years	147	38.2
	14–15 years	166	43.1
	16 years and above	72	18.7
Gender	Male	87	22.6
	Female	298	77.4
Grade of Study	6th grade	13	3.4
	7th grade	120	31.2
	8th grade	78	45.2
	9–10th grade	174	45.2
City of Residence	Rawalpindi	196	50.9
	Islamabad	189	49.1
School Area	Rural	110	28.6
	Urban	275	71.4
Family Members	1–6 members	260	67.5
	7–12 members	105	27.3
	13–18 members	17	4.4
	19 or more members	3	0.8
Family Type	Joint	142	36.9
	Nuclear	243	63.1
Father’s Occupation	Business/self-employed	138	35.8
	Government job	68	17.7
	Private job	155	40.3
	Retired/unemployed	13	3.4
	Late	11	2.9
Mother’s Occupation	Housewife	344	89.4
	Employed	36	9.4
	Late	5	1.3
Income	Less than 50,000/-	175	45.5
	50,000/- –100,000/-	125	32.5
	More than 100,000/-	85	22.1
Parent’s Marital Status	Married	363	94.3
	Separated/divorced	10	2.6
	Widowed	12	3.1
Family History of Gastrointestinal Issues	Yes	173	44.9
	No	212	55.1
Dining Out Frequency	Once a week	143	37.1
	Once every 2 weeks	59	15.3
	Once a month	75	19.5
	Every 2 months	108	28.1
Bringing Home Lunch	Everyday	99	25.7
	Every other day	25	6.5
	Once a week	50	13
	Less than once a week	211	54.8
Engaging in Sports/Physical Activity	Everyday	145	37.7
	Every other day	41	10.6
	Once a week	119	30.9
	Less than once a week	80	20.8

**Table 2:** Food Consumption Frequency Data for Children Enrolled In the Study (*N* = 385).

Consumption Frequencies					
Never *n* (%)	Less than Once a Week *n*(%)	Once a Week *n*(%)	2–3 Times a Week *n* (%)	4–6 Times a Week *n*(%)	Everyday *n*(%)
**Cereals**					
0 (0)	17 (4.4)	131 (34)	195 (50.6)	40 (10.4)	2 (0.5)
**Fruits**					
3 (0.8)	57 (14.8)	120 (31.2)	115 (29.9)	55 (14.3)	35 (9.1)
**Vegetables**					
8 (2.1)	129 (33.5)	161 (41.8)	65 (16.9)	14 (3.6)	8 (2.1)
**Meat and Meat Compounds**					
2 (0.5)	92 (23.9)	166 (43.1)	90 (23.4)	22 (5.7)	13 (3.4)
**Pulses and Legumes**					
26 (6.8)	112 (29.1)	141 (36.6)	73 (19.0)	26 (6.8)	7 (1.9)
**Dairy and Dairy Products**					
3 (0.8)	65 (16.9)	93 (24.2)	118 (30.6)	70 (18.2)	36 (9.4)
**Snacks**					
0 (0)	32 (8.3)	76 (19.7)	122 (31.7)	107 (27.8)	48 (12.5)
**Beverages**					
2 (0.5)	30 (7.8)	96 (24.9)	127 (33)	93 (24.2)	37 (9.6)

**Table 3:** Prevalence Data for Functional Gastrointestinal Symptoms in Children Enrolled in the Study (*n* = 385).

FGID Absent *n* (%)	FGID Present *n* (%)
**Post-prandial Distress Syndrome**	
366 (95.1)	19 (4.9)
**Epigastric Pain**	
344 (89.4)	41 (10.6)
**Irritable Bowel Syndrome**	
289 (75.1)	96 (24.9)
**Abdominal Migraine**	
382 (99.2)	3 (0.8)
**Functional Abdominal Pain**	
236 (61.3)	149 (38.7)
**Functional Constipation**	
331 (86)	54 (14)
**Functional Nausea**	
321 (83.4)	64 (16.6)
**Functional Vomiting**	
383 (99.5)	2 (0.5)
**Adolescent Rumination Syndrome**	
379 (98.4)	6 (1.6)
**Aerophagia**	
381 (99)	4 (1)

**Table 4:** Association of Functional Gastrointestinal Diseases with Socio-Demographic and Dietary Factors.

Variables	Categories	With Post-Prandial Distress *n* (%)	With Epi-Gastric Pain *n* (%)	With Irritable Bowel Syndrome *n* (%)	With Functional Abdominal Pain *n* (%)	With Functional Nausea *n* (%)	With Abdominal Migraine *n* (%)	With Adolescent Rumination Syndrome *n* (%)
Age	12–13 years	10 (52.6)	16 (39.0)	38 (39.6)	66 (44.3)	27 (42.2)	1 (33.3)	1 (16.7)
	14–15 years	6 (31.6)	16 (39.0)	34 (35.4)	65 (43.6)	26 (40.6)	1 (33.3)	2 (33.3)
	16 years and above	3 (15.8)	9 (22.0)	24 (25.0)	18 (12.1)	11 (17.2)	1 (33.3)	3 (50.0)
	***p*-value**	0.43	0.80	0.10	0.01 *	0.76	0.59	0.13
Gender	Male	3 (15.8)	16 (39.0)	10 (10.4)	47 (31.5)	12 (18.8)	0 (0.0)	2 (33.3)
	Female	16 (84.2)	25 (61.0)	86 (89.6)	102 (68.5)	52 (81.3)	3 (100.0)	4 (66.7)
	***p*-value**	0.58	0.008 *	0.001 *	0.001 *	0.42	1.00	0.52
Number of Family Members	1–6 members	14 (73.7)	25 (61.0)	60 (62.5)	104 (69.8)	47 (73.4)	2 (66.7)	4 (66.7)
	7–12 members	4 (21.1)	14 (34.1)	26 (27.1)	38 (25.5)	11 (17.2)	1 (33.3)	2 (33.3)
	13–18 members	1 (5.3)	1 (2.4)	9 (9.4)	6 (4.0)	6 (9.4)	0 (0.0)	0 (0.0)
	19 or more members	0 (0)	1 (2.4)	1 (1.0)	1 (0.7)	0 (0.0)	0 (0.0)	0 (0.0)
	***p*-value**	0.73	0.31	0.05 *	0.91	0.04 *	1.00	1.00
Income	Less than 50,000/-	6 (31.6)	9 (22.0)	41 (42.7)	74 (49.7)	25 (39.1)	1 (33.3)	1 (16.7)
	50,000/- to 100,000/-	4 (21.1)	16 (39.0)	33 (34.4)	47 (31.5)	25 (39.1)	2 (66.7)	3 (50.0)
	More than 100,000/-	9 (47.4)	16 (39.0)	22 (22.9)	28 (18.8)	14 (21.9)	0 (0.0)	2 (33.3)
	***p*-value**	0.04 *	0.002 *	0.82	0.33	0.42	0.60	0.36
Dining Out Frequency	Once a week	8 (42.1)	17 (41.5)	36 (37.5)	54 (36.2)	19 (29.7)	3 (100.0)	2 (33.3)
	Once every 2 weeks	3 (15.8)	12 (29.3)	17 (17.7)	23 (15.4)	9 (14.1)	0 (0.0)	2 (33.3)
	Once a month	2 (10.5)	6 (14.6)	18 (18.8)	31 (20.8)	17 (26.9)	0 (0.0)	2 (33.3)
	Every 2 months	6 (31.6)	6 (14.6)	25 (26.0)	41 (27.5)	19 (29.7)	0 (0.0)	0 (0.0)
	***p*-value**	0.79	0.02 *	0.87	0.96	0.35	0.31	0.30
Bringing Home Lunch	Everyday	6 (31.6)	5 (12.2)	24 (25.0)	47 (31.5)	17 (26.6)	1 (33.3)	1 (16.7)
	Every other day	0 (0.0)	1 (2.4)	9 (9.4)	12 (8.1)	1 (1.6)	0 (0.0)	1 (16.7)
	Once a week	0 (0.0)	9 (22.0)	13 (13.5)	17 (11.4)	9 (14.1)	2 (66.7)	0 (0.0)
	Less than once a week	13 (68.4)	26 (63.4)	50 (52.1)	73 (49.0)	37 (57.8)	0 (0.0)	4 (66.7)
	***p*-value**	0.204	0.04 *	0.60	0.10	0.36	0.04 *	0.54
Engaging in Sports/Physical Activity	Everyday	4 (21.1)	5 (12.2)	30 (31.3)	75 (50.3)	21 (32.8)	1 (33.3)	2 (33.3)
	Every other day	1 (5.3)	1 (2.4)	11 (11.5)	15 (10.1)	10 (15.6)	0 (0.0)	2 (33.3)
	Once a week	4 (21.1)	9 (22.0)	26 (27.1)	34 (22.8)	19 (29.7)	1 (33.3)	2 (33.3)
	Less than once a week	10 (52.6)	26 (63.4)	29 (30.2)	25 (16.8)	14 (21.9)	1 (33.3)	0 (0.0)
	***p*-value**	0.01 *	0.83	0.05 *	0.001 *	0.50	1.00	0.23
Fruits Consumption	Never	0 (0.0)	0 (0.0)	1 (1.0)	1 (0.7)	0 (0.0)	0 (0.0)	0 (0.0)
	Less than once a week	8 (42.1)	5 (12.2)	15 (15.6)	25 (16.8)	10 (15.6)	0 (0.0)	0 (0.0)
	Once a week	7 (36.9)	8 (19.5)	31 (32.3)	45 (30.2)	21 (32.8)	1 (33.3)	3 (50.0)
	2–3 times a week	1 (5.3)	13 (31.7)	29 (30.2)	41 (27.5)	20 (31.3)	2 (66.7)	3 (50.0)
	4–6 times a week	3 (15.8)	9 (22.0)	12 (12.5)	18 (12.1)	5 (7.8)	0 (0.0)	0 (0.0)
	everyday	0 (0.0)	6 (14.6)	8 (8.3)	19 (12.8)	8 (12.5)	0 (0.0)	0 (0.0)
	***p*-value**	0.007 *	0.28	0.98	0.33	0.56	0.91	0.56
Vegetables consumption	Never	0 (0.0)	0 (0.0)	2 (2.1)	4 (2.7)	1 (1.6)	0 (0.0)	0 (0.0)
	Less than once a week	11 (57.9)	11 (26.8)	34 (35.4)	55 (36.9)	29 (45.3)	1 (33.3)	3 (50.0)
	Once a week	5 (26.3)	21 (51.2)	37 (38.5)	58 (38.9)	27 (42.2)	2 (66.7)	3 (50.0)
	2–3 times a week	1 (5.3)	6 (14.6)	18 (18.8)	22 (14.8)	5 (7.8)	0 (0.0)	0 (0.0)
	4–6 times a week	0 (0.0)	3 (7.3)	4 (4.2)	5 (3.4)	2 (3.1)	0 (0.0)	0 (0.0)
	everyday	2 (10.5)	0 (0.0)	1 (1.0)	5 (3.4)	0 (0.0)	0 (0.0)	0 (0.0)
	***p*-value**	0.03 *	0.46	0.92	0.49	0.11	1.00	0.18
Pulses and lentil consumption	Never	3 (15.8)	3 (7.3)	3 (3.1)	17 (11.4)	2 (6.3)	0 (0.0)	1 (16.7)
	Less than once a week	5 (26.3)	13 (31.7)	25 (26.0)	40 (26.8)	19 (29.7)	1 (33.3)	0 (0.0)
	Once a week	9 (47.4)	15 (36.6)	37 (38.5)	56 (37.6)	27 (42.2)	0 (0.0)	1 (16.7)
	2–3 times a week	1 (5.3)	8 (19.5)	21 (21.9)	22 (14.8)	11 (17.2)	1 (33.3)	2 (33.3)
	4–6 times a week	1 (5.3)	0 (0.0)	8 (8.3)	11 (7.4)	2 (3.1)	1 (33.3)	2 (33.3)
	everyday	0 (0.0)	2 (4.9)	1 (1.0)	3 (2.0)	1 (1.6)	0 (0.0)	0 (0.0)
	***p*-value**	0.35	0.26	0.31	0.05 *	0.85	0.20	0.05 *
Beverages consumption	Never	0 (0.0)	1 (2.4)	0 (0.0)	0 (0.0)	0 (0.0)	0 (0.0)	0 (0.0)
	Less than once a week	1 (5.3)	3 (7.3)	7 (7.3)	13 (8.7)	1 (1.6)	0 (0.0)	0 (0.0)
	Once a week	9 (47.4)	5 (12.2)	22 (22.9)	40 (26.8)	15 (23.4)	1 (33.3)	0 (0.0)
	2–3 times a week	4 (21.1)	18 (43.9)	37 (38.5)	43 (28.9)	22 (34.4)	0 (0.0)	2 (33.3)
	4–6 times a week	5 (26.3)	11 (26.8)	22 (22.9)	34 (22.8)	16 (25.0)	0 (0.0)	4 (66.7)
	everyday	0 (0.0)	3 (7.3)	8 (8.3)	8 (12.8)	10 (15.6)	2 (66.7)	0 (0.0)
	***p*-value**	0.23	0.12	0.87	0.35	0.18	0.04 *	0.19

All values marked with an asterisk (*) indicate statistically significant associations between the variables.

## Data Availability

Data is available upon editor’s request.
